# Apple allergy: Causes and factors influencing fruits allergenic properties–Review

**DOI:** 10.1002/clt2.12032

**Published:** 2021-06-02

**Authors:** Aleksandra Siekierzynska, Dorota Piasecka‐Kwiatkowska, Aleksander Myszka, Marta Burzynska, Barbara Sozanska, Tomasz Sozanski

**Affiliations:** ^1^ Department of Physiology and Plant Biotechnology Institute of Agricultural Sciences, Land Management and Environmental Protection University of Rzeszow Rzeszow Poland; ^2^ Department of Food Biochemistry and Analysis Poznan University of Life Sciences Poznan Poland; ^3^ Institute of Medical Sciences University of Rzeszow Rzeszow Poland; ^4^ 1st Department of Pediatric Allergology and Cardiology Wroclaw Medical University Wroclaw Poland; ^5^ Department of Pharmacology Wroclaw Medical University Wroclaw Poland

**Keywords:** allergy, antioxidants, apple varieties, birch pollinosis, polyphenols

## Abstract

**Background:**

Apple tree fruits (*Malus* × *domestica* Borkh.) are a rich source of nutrients and nutraceuticals and are recommended as a part of the healthy, staple diet. However, apples could be also the cause of allergies including severe reactions. Allergies to fruits like apples are predominantly associated with pollinosis. In North and Central Europe, sensitisation to apples is caused mainly by cross‐reactive birch pollen aeroallergen, whereas in the Mediterranean area of Europe, apple allergy is mostly associated with allergies to peach. The allergenicity of apples differ across cultivars but only a few varieties were studied. Some factors changing apples allergenicity were identified, including unmodifiable and potentially modifiable factors for example cultivation method, ripening stage and storage conditions.

**Aim:**

This review presents current knowledge about the molecular basis of apple allergenicity and factors influencing its level.

**Conclusions:**

Selecting cultivars with low potential of allergenicity, removing apple peel and heat treatment could reduce the risk of severe allergy reaction incidence and presumably can be used in birch pollen immunotherapy.

**Abbreviations**
BAT: basophil activation test
BMI: body mass index
CCDs: cross reactive carbohydrate determinants
EAST: Enzyme Allergosorbent Test
ELISA: Enzyme‐Linked Immunosorbent Assay
EST: expressed sequence tag
HR: histamine release test
LG: linkage group
(ns)LTPs: (non‐specific) lipid transfer proteins family
OAS: oral allergy syndrome
OPT: oral provocation test
PPO: polyphenol oxidase enzyme
PR: Pathogen Resistance Proteins; pathogenesis‐related protein family
PR: Pathogen Resistance Proteins; pathogenesis‐related protein family
R: resistant
RAST: inhibition and immunoblotting
S: susceptible
sIgE: specific IgE
SPT: skin prick test
VS: very susceptible

## 1 BACKGROUND

Apple tree fruits (*Malus* × *domestica* Borkh.) are a rich source of nutrients and nutraceuticals like polyphenols and other phytochemicals. The main components of apple phytochemicals are phenolic acids, dihydrochalcones, flavonoids (quercetin glycosides), catechins and oligomeric procyanidins as well as cyanidin glucosides in red fruits.^1^ Due to the listed ingredients, apple may reduce risk of chronic diseases, through various mechanisms, including antioxidant or anti‐proliferative.^2^ They may also improve the functioning of the digestive tract, regulate body mass and increase the respiratory efficiency of the body.^3^ Unfortunately, apples could also be the cause of allergies including severe reactions.

About 5%–8% children and 2%–3% adults suffer from food allergy.^4^ Allergies to fruits like apple, pear, peach, apricot, cherry, and to vegetables such as carrot, celery and potato are more frequent in older children and adults and they are predominantly associated with cross‐reactivity between aeroallergens like tree pollens, grass or ragweed pollens and food allergens due to structural homology of some allergenic proteins.^5^ In North and Central Europe, the most frequent example is the symptomatic response to raw apple in patients sensitised to birch tree pollens.^6^ The primary sensitisation to allergenic molecules of *Betula verrucosa* (e.g., Bet v1) triggers the synthesis of specific IgE antibodies which are capable to cross‐react with its homologues in apple (e.g., Mal d1). The clinical expression of such immune‐mediated reaction includes rapid‐onset pruritus of the oropharynx, angio‐oedema, ears' pruritus and sometimes larynx constriction. These symptoms known as an Oral Allergy Syndrome (OAS) are usually mild and occur directly after exposure to the allergens. The apple allergens are heat‐labile and susceptible to digestion thus the symptoms are rarely connected with gastrointestinal track. Asero et al.^7^ estimated the pathogenesis‐related protein family PR‐10 and profilin are although labile molecules, can induce systemic reactions facilitated by proton pump inhibitors, ingestion of large amounts of raw foods and fasting. The cross‐reactivity properties and allergenic potential of different apple cultivars may vary and this phenomenon may be clinically useful in planning oral immunotherapy treatment with the use of less allergenic cultivars. These issues will be discussed in our article.

### 1.1 Sensitising components

#### 1.1.1 Mal d proteins

So far, four allergens have been identified and officially incorporated into the nomenclature by WHO/IUIS^8^ in apples (*Malus* × *domestica* Borkh.): Mal d 1, Mal d 2, Mal d 3 and Mal d 4. Among them, Mal d 1 is clinically the most important allergen in North and Central Europe, Mal d 3 in Southern Europe. In Mediterranean, the two other proteins Mal d 2 and Mal d 4, are also associated with the hyper‐reactivity to apple fruits.

Mal d 1 is identified as a 17‐18 kDa protein of 158‐159 amino acids encoded by 480‐483 nucleotides.^9^ Its biological function is connected with fungal and bacterial infection response due to the ribonuclease activity of proteins belonging to the pathogenesis‐related protein family (PR‐10). Mal d 1 may also be involved in binding and transport of plant steroids and intracellular signalling.^10^, ^11^, ^12^ The abiotic and biotic stress affects the content of Mal d 1 allergen. Time and conditions of apple fruits storage may quantitatively alter the allergenic properties of their proteins.^13^ Moreover, patients with birch pollen‐related food allergies report the severity of their symptoms strongly dependent on apple variety and the degree of maturity.^14^ Variability in the allergenic potency might result from the different expression levels of the Mal d 1 isoforms clustered in four groups (Mal 1.01–Mal d 1.04) (www.allergen.org). In the apple genome, many sequences of isoallergens has been identified so far. Mal d 1 is encoded by 18 genes, seven of these are clustered into linkage group 13 (LG13), nine genes clustered into LG16 and one of them is unclustered.^9^ The gene family was divided into five groups depending on number and size of introns and analysis of EST (expressed sequence tag).^15^ In the first subfamily of the Mal d 1 protein, two major genetic variants *Mal d 1.01* and *Mal d 1.02*, in the second *Mal d 1.04* and *Mal d 1.05*, in the third *Mal d 1.06A*, *Mal d 1.06B*, *Mal d 1.06C*, in the fourth: *Mal d 1.07‐1.09*, *Mal d 1.03A‐G* and in the fifth *Mal d 1ps1* have been identified.^9^ Gao et al.^16^ demonstrated the association of expression of *Mal d 1.04* and *Mal d 1.06A* with the allergenicity. Moreover, *Mal d 1.06A* showed the allele dosage effect on the amount of Mal d 1 protein.^16^

Another apple allergen Mal d2 (23 kDa), is also known to be connected with apple allergenicity phenomenon. Mal d 2 belongs to thaumatin‐like proteins (TLPs) group with antifungal properties (PR‐5).^17^ TLPs are major protein component in mature apple fruit^18^ and they are considered as a panallergen in food and in pollen.^19^ Mal d 2 is similar to protein extracted from the fruit *Thaumatococcus daniellii*. Mal d 2 proteins are encoded by *Mal d 2.01, 2.02* and *2.03* genes,^20^ although there is only one isoform (Mal d 2.01) officially recognised by WHO/IUIS.^8^ Two copies of the *Mal d 2.01* gene are slightly different in the signal peptide and intron size mapped at the same position on LG 9.^21^ Mal d 2 proteins are very stable molecules, resistant to heat denaturation and proteolysis, as a result of the presence of the eight disulphide bridges which hold together three‐dimensional structure.^22^ Hsieh et al.^23^ identified Mal d 2 as an in vitro reactive allergen among 75% (25/34) of apple allergic subjects recruited in the study in USA.^23^

A 9 kDa molecular weight protein—Mal d 3 identified in apples, belongs to the non‐specific lipid transfer proteins family (nsLTPs). Proteins from that family are major allergens sensitising patients with non‐pollen related allergies to *Rosaceae* fruits.^24^, ^25^, ^26^, ^27^ In Mediterranean countries, patients allergic to apples, but not sensitised to *Betula* pollen, confer allergies to peach and other *Rosaceae* and non‐*Rosaceae* fruits. Apple Mal d 3 allergens cross‐react with peach Pru p 3 allergens. Mal d 3 is encoded by two genes *Mal d 3.01* and *Mal d 3.02*.^25^

Mal d 4 is a cytosolic protein 12–15 kDa,^24^ playing essential role in plant growth and development by participating in the regulation of actin filament polymerisation.^20^ Allergy to Mal d 4 occurs mostly in the Mediterranean, with minor role in apple sensitisation.^26^ This allergen is involved in sensitisation to fruits of other species and strongly cross‐reacts with birch pollen Bet v 2 profilin.^13^ ^,^ ^28^ Mal d 4 is encoded by three genes *Mal d 4.01, Mal d 4.02* and *Mal d 4.03*,^20^ among them *Mal d 4.02* has the highest expression level.^21^

### 1.2 CCDs—cross reactive carbohydrate determinants

About 20% of sensitised patients to pollen produce IgE antibodies that can bind carbohydrate determinants. IgE specific to CCDs are considered to have no or minor clinical significance, Glycans with carbohydrate determinants in plants and in invertebrates differ from those glycoproteins existing in mammals. These foreign epitopes for humans are highly immunogenic resulting in specific IgE antibodies.^29^ The widespread presence of fucose and xylose on N‐linked glycans of plants and in invertebrates may explain the high degree of cross‐reactivity that has been reported for CCD‐specific IgE antibodies.^30^ The clinical relevance of IgE antibodies to CCDs relies on the composition of the allergen‐monovalent or multivalent with respect to the carbohydrate determinant.

To avoid misdiagnosis, an investigation of the presence of CCD antibodies should be conduct. Determination of anti‐CCD IgE antibody in blood can be tested with bromelain or horseradish peroxidase, and also by the use of a test specific to MUXF3, a common plant glycan structure.^31^ A positive in vitro test and a negative skin prick test to the same plant food allergen may indicate presence of non‐cross‐linking CCD‐specific IgE antibodies to that allergen. However, this phenomenon does not exclude cross‐linking to other allergens with multivalent CCD epitopes or the presence of concomitant IgE antibodies to peptide epitopes.^32^

### 1.3 Allergenicity varies regards to apple tree varieties and cultivation method

Despite common allergies to apples, only a few studies assessing the amount of different apple allergens were conducted in commonly cultivated varieties: Golden Delicious, Granny Smith, Fuji, Santana, Cox's Orange Pippin, Topaz, Braeburn^12^ ^,^ ^30^ ^,^ ^31^ and mainly in relation to the Mal d 1 (Tables 1 and 2). The extensive use of these popular cultivars has resulted in uniformity of commercial apple orchards and the limitation of genetic biodiversity.^33^

**TABLE 1:** The list of apple cultivars and methods used for Mal d allergens studies

Cultivar	Analysis method	Mal d 1	Mal d 2	Mal d 3	Mal d 4
Golden Delicious	Gene expression	3, 21, 34, 35, 36	21, 34, 35, 36	21, 35, 36, 37	21, 38
	ELISA, EAST, immunoblotting	3, 14, 16, 21, 34, 36, 38, 39, 40, 41, 42, 48, 49, 50	21, 34	21, 42, 43	21, 38
	SPT/prick‐to‐prick	36, 41, 44	36	43	21, 35
Granny Smith	Gene expression	3, 21, 36	21	21	21
	ELISA, EAST, immunoblotting	3, 14, 16, 31, 34, 42, 48, 50	36	43	
	SPT/prick‐to‐prick	44		43	
Baeburn	Gene expression	21	21	21	21
	ELISA, EAST, immunoblotting	13, 14, 21, 35, 38, 39, 40	21	21	21
	SPT/prick‐to‐prick	13			
Elstar	Gene expression	3			
	ELISA, EAST, immunoblotting	3			38
	SPT/prick‐to‐prick				38
Topaz	Gene expression				
	ELISA, EAST, immunoblotting	13, 39, 40, 45			
	SPT/prick‐to‐prick				
Elise	Gene expression				
	ELISA, EAST, immunoblotting	13, 45			
	SPT/prick‐to‐prick	44			
Santana	Gene expression	46			
	ELISA, EAST, immunoblotting	49			
	SPT/prick‐to‐prick	16, 44			
Cox's Orange Pippin, Jonagored, Jonagold, Boskoop, Priscilla, Fuji, Jonathan, prima, Fiesta, Mcintosh, Gala, Idared, Gloster, Szampion	Gene expression	3, 36, 47			
	ELISA, EAST, immunoblotting	3, 14, 28, 36, 38, 40, 45, 47, 50		43	
	SPT/prick‐to‐prick	16, 41, 44		43	
Old varieties: Pink Lady Cripps Pink, Annurca, ‘Calvilla Bianca d’Inverno, Mutsu, Osnabruecker Renette, Delorina, Resista, Rajca, Grey Renette, Starking	Gene expression,	21, 36	21, 36	21, 36	21
	ELISA, EAST, immunoblotting	21, 36	21, 36	43	21
	SPT/prick‐to‐prick	40,41,44	43	43	

Abbreviations: EAST, Enzyme Allergosorbent Test; ELISA, enzyme‐linked immunosorbent assay; SPT, Skin Prick Test.

**TABLE 2:** Mal d 1 protein content across apple cultivars

Cultivar	Mal d 1 content	Units	Literature
Golden Delicious	12.1	µg/g FW	3
	C_50_ = 0.12, C_50_ = 0.36	µg	14
	45 (4.5)	µg/g (mg/100 g)	48
	2.9, 3.4, about 10.0	µg/g FW	49
	14.1–135.17	µg/g	38
	7.3–18.6	µg/g FW	16
	6.2–7.6	µg/g f FW	40
	7–8 (0.7–0.8)	µg/g (mg/100 g)	34
	7.6–17	µg/g	39
	5.5–12.8	µg/g	50
Granny Smith	5.95–18.17	µg/g	16
	12.14, 8.81	µg/g FW	3
	16 (1.6)	µg/g (mg/100 g)	48
	2.3–6.4	µg/g FW	34
	5.45–12.14	µg/g	50
Baeburn	9.45–271.20	µg/g	38
	C_50_ = 0.12	µg	14
Topaz	2.0–6.4	µg/g FW	40
	6.3–16.1	µg/g	39
	<1–25	µg/g FW	45
Santana	0.5, 2.3, about 5.0	µg/g FW	49
Elise	0.25–17	µg/g FW	45
Fuji	11.50	µg/g FW	3
	5.4, 11.5	µg/g	28
	32.84–455.01	µg/g	38
	50.8	µg/g	47
Boskoop	C_50_ = 2	µg	14
	1–25	µg/g FW	45
Jonagold	7 (0.7)	µg/g (mg/100 g)	48
	3.33–5.5	µg/g FW	50
	1.3–8.7	µg/g FW	40
	12.98–38.82	µg/g	38
	17.2	µg/g	47
Idared	8 (0.8)	µg/g (mg/100 g)	48
Gloster	4 (0.4)	µg/g (mg/100 g)	48
Gala	14.6	µg/g FW	40

Abbreviations: C50, concentration of protein causing 50% inhibition of IgE binding from patients sera; FW, fresh weight.

The issue of the expression of genes encoding allergens in apples is also poorly understood. Even limited number of publications regarding the gene expression encoding apple allergens showed an association among gene expression and degree of maturity, storage conditions mainly with respect to the *Mal d 1* gene (Table 1). Therefore, it is expected to expand similar research to a larger number of varieties, in particular with regard to old ones. There are only a few papers^40^ ^,^ ^44^ describing allergen gene expression in old varieties, which are valuable in terms of taste, nutritional value, processing or for breeding. Nevertheless, cultivation of apple varieties with low allergenic potential is not developed. Currently, only Santana, Topaz and Elise cultivars, are considered hypoallergenic, and are quite well tolerated by patients with allergies. It has been shown that the Santana variety is characterised by considerable resistance to apple scab, thanks to which it is possible to significantly reduce the use of fungicides in its cultivation.^51^ There is some evidence that pesticide treatment may lead to an even more robust response inducing higher expression of Mal d 1 than biotic factor.^40^

### 1.4 Abiotic factors influencing allergenicity

Variation of Mal d 1 content during ripening, postharvest maturity, and storage time and conditions were confirmed only in the context of Golden Delicious, Topaz, Braeburn and Cox’Orange Pippin apple varieties. During the ripening period, content of Mal d 1 allergens continuously increases from about 0.2 mg/100 g fresh weight to approximately 0.8 mg/100 g fresh weight (130–164 days after blooming respectively). Mal d 1 content in apple fruit varieties Braeburn, Topaz harvested at different stages of ripeness revealed no differences.^36^ After an additional shelf life, significantly higher Mal d 1 concentration in the overripe fruit in comparison to the unripe fruit were determined. Storage, at ambient temperature, of 12 weeks cold‐stored fruit of above mentioned cultivars led also to Mal d 1 accumulation in unripe and overripe harvested fruit, contrary to ripe fruit, where Mal d 1 remained stable.^39^ The study emphasises the need for further research on other apple cultivars to ascertain the differences of Mal d 1 content at different maturity stage and during apple storage. Several studies shown the elevation of Mal d 1 protein content and up‐regulation of *Mal d 1* gene expression during storage and by cold stress.^40^ However, Botton et al.^21^ indicated stable level of gene expression in analysed apple cultivars, inter alia in Golden Delicious and Braeburn. *Mal d 3* expression was two to five times higher in apple skin than in a pulp and down‐regulated upon storage time by about 5 months. The duration of storage time down regulates Mal d 4 coding genes.^21^ Yang et al.^35^ showed a decrease in expression of the Mal d 4 isoforms after harvest and during ripening. It is suggested that different responses to ethylene can affect profilin gene expression.

A point of interest is that organic farming weakened Mal d content,^52^ unfortunately, commercial varieties with a significantly reduced resistance to apple diseases are not suitable for this type of crop. We should focus our attention on old varieties, in which a significant resistance to fungal diseases is observed, making them suitable for organic farming that is getting modern nowadays. Moreover, the impact of cultivation methods on allergenicity is not established nor in commonly cultivated apple trees neither in old cultivars.

### 1.5 Biotic factors

Plants react to pathogen attacks (Table 3), wounding, UV‐B radiation, osmotic shock, low temperature, water deficit, chemicals like ethylene or salicylic acid, inter alia by producing proteins belonging to the PR (Pathogen Resistance Proteins) family. Three of the four main apple allergens belongs to PR, which are connected with natural resistance to powdery mildew or/and to apple scab or to other stressors and chemicals.^53^

**TABLE 3:** Fungi disease susceptibility and allergenicity

Apple variety	Apple scab	Powdery mildew	Allergenicity	Study
Golden delicious	S	VS	High	54
Granny smith	S	VS	Low	55
Braeburn	R	R	Low/high	14,55
Elstar	S	S	Low	55
Topaz	R	R	Low	55
Elise	S/R	S	Low	55
Santana	R	S	Low	55

Abbreviations: R, resistant; S, susceptible; VS, very susceptible.

### 1.6 Allergenicity modifying factors

The allergenicity of apples is more complex due to the interactions of Mal d 1 protein with polyphenols (catechin) and enzymatic antioxidant system. The reaction between Mal d 1 and oxidised polyphenols can result in decrease of IgE binding as shown in Braeburn cultivar.^39^ On the other hand, in Topaz, with high polyphenols content and low activity of PPO (polyphenol oxidase enzyme) conferring a high total anti‐oxidative capacity, IgE binding to Mal d 1 is also reduced.^56^ According to Schmits‐Eiberger and Matthes,^39^ in the cv. Braeburn, cv. Golden Delicious and cv. Topaz amount of total polyphenols were stable during maturation; however during storage, polyphenol content significantly decreased.

Traditional cultivation of apple varieties with low allergenic potentials is not well developed. Currently, Santana, Topaz and Elise cultivars, are considered as hypoallergenic, and are quite well tolerated in patients experiencing OAS syndrome.^40^ Furthermore, it has been demonstrated that Santana is characterised by considerable resistance to apple scab, thanks to which it is possible to significantly reduce the usage of chemicals with anti‐fungal properties in its cultivation,^51^ thus the use of organic cultivation of this variety may led to reduce the amount of allergenic proteins. In a recent study of allergenic potential of apples cultivars, tested by prick‐to‐prick skin tests and provocation test in 52 patients with birch pollen hay fever and OAS, significant differences among various cultivars were revealed. Red‐fleshed cultivars gave the mildest reactions, being proposed as potentially useful tool in oral immunotherapy treatment in patients with birch pollen allergy and OAS due to birch‐apple cross‐reactivity.^57^ Post‐harvest treatment may have additional role in apple fruit allergenicity. Hsieh et al.^23^ revealed that prolonged storage at 4°C of Golden Delicious and Granny Smiths fruits can elevate Mal d 1 and Mal d 2 protein levels. In low allergenic cultivars like Santana and Elise, Mal d 1 proteins increased along with storage time, but after treatment with MCP‐1‐inhibiting ripening, the content of Mal d 1 protein was reduced.^45^

## 2 CONCLUSIONS

In Northern and Central Europe, apple allergies are mostly related to birch pollen sensitisation and are caused by cross‐reactive proteins Bet v 1 and Mal d 1. In the Mediterranean, apple allergies are less frequent but severe and associated with sensitisation to LTPs (Mal d 3).

Variation in the Mal d 1 isoforms expression may account for the variability of allergenic potency of apple cultivars, which suggests that genetic factors could have a major role in controlling the Mal d 1 allergenicity in mature apples.

The differences in the allergenic potential of apple fruits can be also the effect of the degree of ripeness of the fruit, as a result of an accumulation of Mal d 1 protein during maturation. Similarly, the time and conditions of fruit storage affect the accumulation of the Mal d 1 and Mal d 2 allergens as shown in Golden Delicious and Granny Smiths varieties.

The total anti‐oxidative status of apple fruits and interactions of polyphenols with Mal d 1 protein can affect the allergenic potential and the ability to bind IgE antibodies.

Currently, only the Topaz, Elise and Santana varieties are considered to be well tolerated by apple allergic patients.

Selecting cultivars with low potential of allergenicity, removing apple peel and heat treatment could reduce the risk of severe allergy reaction incidence and presumably can be used in birch pollen immunotherapy. Knowledge of the molecular mechanism of apples allergenicity and factors that modify the reaction severity could facilitate medical counselling and improve patients' care with allergies related with apple fruits.

## CONFLICT OF INTERESTS

The authors declare that there are no conflict of interests.

## AUTHOR CONTRIBUTIONS

Aleksandra Siekierzynska made substantial contributions to conception and design and drafted the manuscript. Aleksandra Siekierzynska, Aleksander Myszka and Marta Burzynska participated in literature study. Dorota Piasecka‐Kwiatkowska, Barbara Sozanska and Tomasz Sozanski revised critically for important intellectual content.
